# Determining the mechanical properties of plectin in mouse myoblasts and keratinocytes

**DOI:** 10.1016/j.yexcr.2014.10.001

**Published:** 2015-02-15

**Authors:** Navid Bonakdar, Achim Schilling, Marina Spörrer, Pablo Lennert, Astrid Mainka, Lilli Winter, Gernot Walko, Gerhard Wiche, Ben Fabry, Wolfgang H. Goldmann

**Affiliations:** a Department of Physics, Friedrich-Alexander-University of Erlangen-Nuremberg, Erlangen, Germany; b Department of Molecular Cell Biology, Max F. Perutz Laboratories, University of Vienna, Austria

**Keywords:** Plectin, Intermediate filaments (IF), Mouse myoblasts and keratinocytes, Cell stretching, Magnetic tweezer rheology, Traction force microscopy, Cell motility

## Abstract

Plectin is the prototype of an intermediate filament (IF)-based cytolinker protein. It affects cells mechanically by interlinking and anchoring cytoskeletal filaments and acts as scaffolding and docking platform for signaling proteins to control cytoskeleton dynamics. The most common disease caused by mutations in the human plectin gene, epidermolysis bullosa simplex with muscular dystrophy (EBS-MD), is characterized by severe skin blistering and progressive muscular dystrophy. Therefore, we compared the biomechanical properties and the response to mechanical stress of murine plectin-deficient myoblasts and keratinocytes with wild-type cells. Using a cell stretching device, plectin-deficient myoblasts exhibited lower mechanical vulnerability upon external stress compared to wild-type cells, which we attributed to lower cellular pre-stress. Contrary to myoblasts, wild-type and plectin-deficient keratinocytes showed no significant differences. In magnetic tweezer measurements using fibronectin-coated paramagnetic beads, the stiffness of keratinocytes was higher than of myoblasts. Interestingly, cell stiffness, adhesion strength, and cytoskeletal dynamics were strikingly altered in plectin-deficient compared to wild-type myoblasts, whereas smaller differences were observed between plectin-deficient and wild-type keratinocytes, indicating that plectin might be more important for stabilizing cytoskeletal structures in myoblasts than in keratinocytes. Traction forces strongly correlated with the stiffness of plectin-deficient and wild-type myoblasts and keratinocytes. Contrary to that cell motility was comparable in plectin-deficient and wild-type myoblasts, but was significantly increased in plectin-deficient compared to wild-type keratinocytes. Thus, we postulate that the lack of plectin has divergent implications on biomechanical properties depending on the respective cell type.

## Highlights

• The intermediate filament-associated protein plectin has divergent biomechanical implications depending on cell/tissue type.
• In plectin^−/−^ myoblasts, cell vulnerability, stiffness, strain and binding strength are lower than in wild-type cells.
• Plectin^−/−^ keratinocytes exhibit higher cell stiffness, binding strength, strain and velocity than wild-type cells.

## Introduction

Plectin, a giant (>500 kDa), highly versatile cytolinker protein, is capable of connecting the different cytoskeletal filament systems (intermediate filaments (IFs), actin filaments, and microtubules) with each other to form network-like arrays. Plectin plays a crucial role by anchoring IFs to organelles and to extracellular matrix (ECM)-adhesion complexes at the cytoplasmic membrane, thereby ensuring mechanical stability [1]. As plectin is expressed in practically all cell types, mutations in the human plectin gene (*PLEC*) result in a pleiotropic phenotype and simultaneously affect several tissues, primarily skin, muscle and nerve [2,3]. Plectin׳s function in strengthening cells against mechanical stress is unequivocally demonstrated by the severe tissue fragility of patients suffering from the most common plectin mutation-associated disease, epidermolysis bullosa simplex with muscular dystrophy (EBS-MD) [4]. In the multilayered epithelium of the skin (epidermis), plectin is an essential component of a specialized multiprotein complex forming hemidesmosomes, which provide mechanical stability to basal epidermal keratinocytes by anchoring keratin IFs to the ECM. Plectin is also prominently expressed in striated and smooth muscle cells, but its importance for the structure and function of muscle became only evident when patients with plectin-related EBS were found to suffer, in addition to skin blistering, from a late onset of muscular dystrophy. In skeletal muscle, distinct plectin isoforms are crucial for the integrity of myofibers by specifically targeting and anchoring desmin IFs to Z-disks, costameres, mitochondria and the nuclear/ER membrane system [5,6]. Recent studies have opened up new perspectives on plectin׳s cytolinker function that go far beyond the originally proposed role as a reinforcing element of the cellular cytoarchitecture, as plectin was shown to serve as a scaffold for various proteins involved in signaling, and to interact with a multitude of direct and indirect binding partners [2].

We hypothesize that a disruption of the plectin-mediated cytoskeletal crosslinking and anchorage to the cytoplasmic membrane-located ECM-adhesion complexes should lead to changes in biomechanical properties in terms of mechanical vulnerability, cell stiffness, dynamics, motility and force production. We used wild-type (plectin^+/+^) and plectin-deficient (plectin^−/−^) mouse myoblasts as well as keratinocytes and compared their mechanical behavior to dissect the differential influence of plectin deficiency in these cell lines. Our data provide evidence that the lack of plectin has divergent implications on cellular biomechanical properties depending on the cell type.

## Materials and methods

### Cells and cell culture

Immortalized (p53-deficient) plectin^+/+^ and plectin^−/−^ mouse myoblasts [7] and keratinocytes [8] were used. Myoblasts were cultured in F-10 growth medium (GibcoBRL) supplemented with 20% FCS, 1.5% penicillin/streptomycin (Biochrom AG) and 0.1% essential growth factor (rhFGF, Promega). Keratinocytes were cultured in basal keratinocyte growth medium without calcium (Lonza) supplemented with 2% calcium-free (Chelex 100-treated) FCS, 1% insulin-transferrin-selenium (Gibco), 0.4% bovine pituitary extract (Lonza) and 5 µM calcium.

### Cell stretching

Cell stretch experiments were carried out on flexible polydimethylsiloxan (PDMS, Sylgard) substrates that were molded into the shape of a cell culture well with 4.0 cm^2^ internal surface [9]. The stretcher device consisted of a linear stage for uniaxial stretch and was driven by a computer-controlled stepper motor. The substrates were coated with 5 µg/ml fibronectin in PBS overnight at 4 °C, and 10.000 cells were seeded 24 h prior to experiments. Uniaxial, cyclic stretching was performed in the incubator under normal cell culture conditions (37 °C, 5% CO_2_, 95% humidity) for 1 h and 0.25 Hz at 30% stretch amplitude [10].

### Magnetic tweezer rheology

We used a magnetic tweezer device as described in Ref. [11]. For measurements, 2×10^5^ cells were seeded overnight in a 35 mm diameter tissue culture dish. Thirty minutes before the experiments, cells were incubated with fibronectin-coated paramagnetic beads of 4.5 µm diameter (Invitrogen). A magnetic field was generated using a solenoid with a needle-shaped core (HyMu80 alloy, Carpenter, Reading, PA). The needle tip was placed at a distance of 20–30 µm from a bead bound to the cell using a motorized micromanipulator (Injectman NI-2, Eppendorf). During measurements, bright-field images were taken by a CCD camera (ORCA ER, Hamamatsu) at a rate of 40 frames/s. The bead position was tracked on-line using an intensity-weighted center-of-mass algorithm. Measurements on multiple beads per well were performed at 37 °C for 1 h, using a heated microscope stage on an inverted microscope at 40× magnification (NA 0.6) under bright-field illumination [10].

### Traction force microscopy

Traction measurements were performed on 6.1% acrylamide/bisacrylamide (ratio 19:1) gels (Young׳s modulus 12.8 kPa, thickness 300 µm) with 0.5 μm green fluorescent beads embedded at the top surface [12,13]. Gels were coated with 5 μg/ml fibronectin at 4 °C overnight. Cells were seeded at a density of 5.000 cells cm^-2^ and incubated under normal growth conditions overnight. During measurements, cells were maintained at 37 °C and 5% CO_2_ in a humidified atmosphere. Cell tractions were computed with an unconstrained fast Fourier traction cytometry method [14] and measured before and after treatment with 80 μM cytochalasin D to relax the traction forces [10].

### Migration assay

Fifty thousand cells were seeded in 35 mm diameter dishes coated with 50 µg/ml collagen type I. After 30 min, cells were placed in a microscope incubation chamber (37 °C, 5% CO_2_, 95% humidity), and phase contrast images were recorded every minute for 2 h (10× magnification). Cell movements were computed using a Fourier-based difference-with-interpolation image analysis [13]. Cells moved spontaneously with a mean square displacement (MSD) that also followed a power law with time, MSD=*D* (*t*/*t*_0_)^*β*,^ where *t*_*o*_ is the time interval of the image recordings (1 min) and the prefactor *D* is the apparent diffusivity. The cell velocity was also determined as follows: $v=\sqrt{D}/t_{0}$.

## Results

### Plectin influences the mechanical vulnerability of myoblasts but not of keratinocytes

**Fig. 1:**
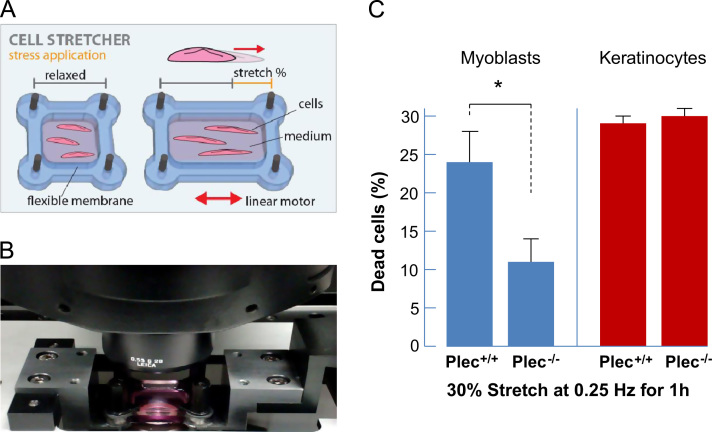
Cell stretcher. Schematic representation (A) and photographic image (B) of the cell stretcher device. Cells were plated on an elastomeric PDMS-membrane coated with the extracellular matrix–protein fibronectin. The cell stretcher was attached to an inverted microscope to observe cell behavior during uniaxial, cyclic stretch. The membrane was stretched up to 30% by a linear motor. (C) Statistical analyses of dead plectin^+/+^ and plectin^−/−^ myoblasts and keratinocytes after cyclic stretch. A total of 40–60 cells were measured per cell type; bars represent mean±SE. ^⁎^*p*<0.05 from unpaired Student׳s *t-*test.

To investigate the response of myoblasts and keratinocytes to mechanical stretch, we cultured cells on flexible PDMS substrates coated with fibronectin and exposed them to uniaxial, cyclic stretch with a peak-to-peak amplitude of maximal 30% at 0.25 Hz for 1 h (Fig. 1A and B). In time-matched control experiments (0% stretch), the percentage of dead cells was around 3% for all cell lines. Increasing the stretch to 10% showed no increase in the number of dead cells, and when the cyclic stretch was further increased to 20%, we observed a slight decrease in overall cell viability (data not shown). However, when we increased the cyclic stretch to 30%, the viability of plectin^+/+^ myoblasts decreased sharply to 23.8% dead cells, compared to only 11.5% of dead cells in plectin^−/−^ myoblasts. By contrast, plectin^−/−^ and plectin^+/+^ keratinocytes showed a similarly high percentage of about 30% of dead cells (Fig. 1C).

### Cellular stiffness of myoblasts and keratinocytes

**Fig. 2:**
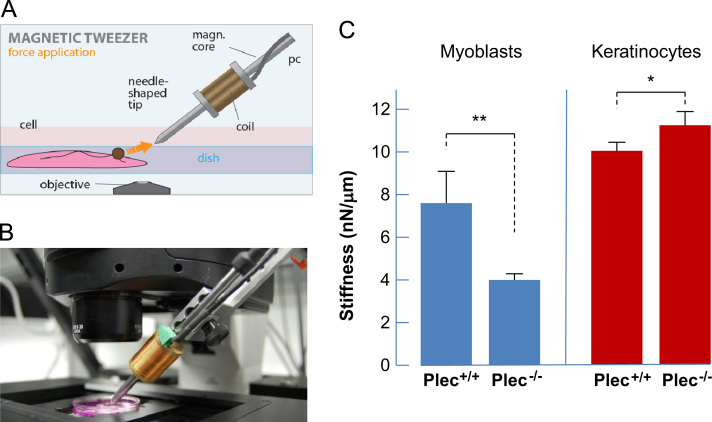
Magnetic tweezer device. Schematic representation (A) and photographic image (B) of the magnetic tweezer setup. A high magnetic field gradient was generated by a needle-shaped solenoid bound to a micromanipulator. The gradient force generated by the magnetic tweezer acted on fibronectin-coated paramagnetic beads. (C) Statistical analyses of cellular stiffness of plectin^+/+^ and plectin^−/−^ myoblasts and keratinocytes. A total of 28–30 cells were measured per cell type; data represent mean±SE. ^⁎^*p*<0.05 and ^⁎⁎^*p*<0.01 from unpaired Student׳s *t-*test.

To measure the cell deformation in response to magnetically generated forces, we performed magnetic tweezer microrheology (Fig. 2A and B). We used this method to apply forces of up to 10 nN to fibronectin-coated superparamagnetic beads attached to myoblasts, or collagen I-coated beads attached to keratinocytes. The bead displacement *d* after a stepwise increase in force *F* followed a power law in time *t* as described in [15]. The cell stiffness was determined from the creep response *J*(*t*) of the cells evaluated at *t*_*o*_=1s, $\;J\left(t\right)=J_{0}\cdot {\left(t/t_{0}\right)}^{\beta }$, where $J_{0}=d(t_{0})/F$, and the stiffness equals 1/*J*_0_. In general, keratinocytes were stiffer than myoblasts (Fig. 2C). Interestingly, the stiffness of plectin^−/−^ myoblasts was twofold lower (~4 nN/µm) compared to plectin^+/+^ myoblasts (~8 nN/µm), while plectin^−/−^ keratinocytes showed a slight increase in stiffness to ~11 nN/µm compared to plectin^+/+^ keratinocytes at ~10 nN/µm.

### Plectin affects binding (adhesion) strength in myoblasts and keratinocytes differently

**Fig. 3:**
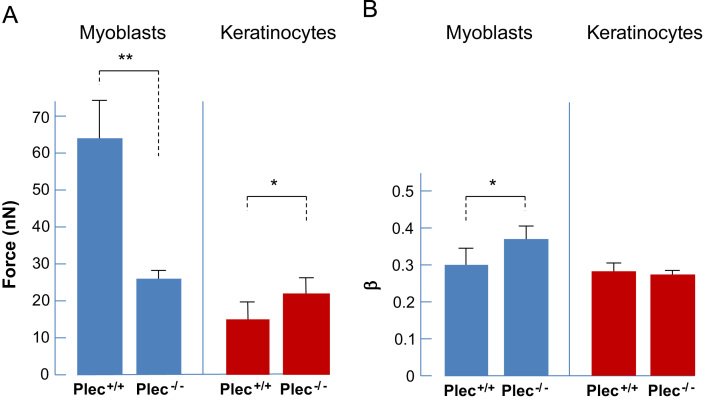
Analyses of bead displacement measurements in response to force ramps up to 80 nN. (A) Statistical analysis of cellular binding (adhesion) strength of plectin^+/+^ and plectin^−/−^ myoblasts and keratinocytes, respectively. (B) Statistical analysis of cell fluidity, that is cellular dynamics (*β*-value). Data in (A) and (B) represent mean±SE; ^⁎^*p*<0.05 and ^⁎⁎^*p*<0.01 from unpaired Student׳s *t-*test. The number of cells analyzed was *n*=26–28 for myoblasts and *n*=54–56 for keratinocytes, respectively.

To quantify the binding strength between ECM-coated beads and cytoskeleton-linked ECM adhesion complexes (focal adhesion complexes and hemidesmosomes), increasing forces of up to 80 nN were applied to the beads using the magnetic tweezer. The median of bead detachment (*p*50 value=median rupture force) was used for the calculation of the binding strength (Fig. 3A). Interestingly, the overall binding strength of myoblasts was considerably higher than that of keratinocytes. Bead detachment was, however, significantly decreased in plectin^−/−^ myoblasts (~26 nN force) compared to plectin^+/+^ myoblasts (~64 nN force). Thus, the binding (adhesion) strength of the integrin–matrix connection was clearly weakened by the loss of plectin (almost 2.5-fold) in myoblasts, while the binding strength of plectin^−/−^ keratinocytes (~20 nN force) was increased compared to their wild-type counterparts (~15 nN force).

To gauge whether the difference in cell stiffness was associated with increased remodeling dynamics of the cytoskeleton, we measured the power law exponent *β* of the creep response (Fig. 3B). A higher power law exponent indicates a more fluid-like behavior of the cell due to a higher dynamics of cytoskeletal components [16]. The fluidity was increased in plectin^−/−^ (*β*=0.35) compared to plectin^+/+^ (*β*=0.29) myoblasts, indicating that plectin stabilizes cytoskeletal structures in these cells. By contrast, the fluidity (i.e., cytoskeletal dynamics) was comparable between plectin^−/−^ and plectin^+/+^ keratinocytes (*β*~0.28).

### Plectin alters contractile force generation in both cell types in a different way

**Fig. 4:**
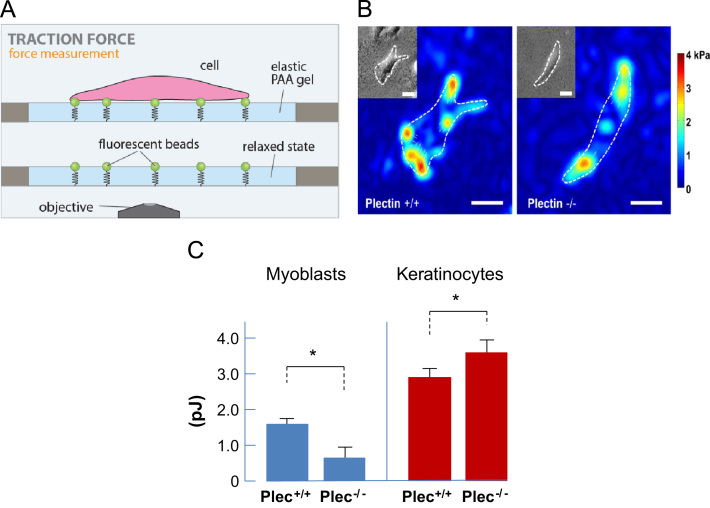
Traction force microscopy. (A) Schematic diagram of the setup. (B) Traction map with bright field image (inset) of a plectin^+/+^ and plectin^−/−^ myoblast, respectively. Bars, 20 µm. (C) Statistical analysis of the strain energy normalized to the spreading area. Cells (*n*=15–21) were measured per cell type; the data represent mean±SE. ^⁎^*p*<0.05 from unpaired Student׳s *t-*test.

The contractile forces of spread cells are predominantly transmitted to the ECM by the actin cytoskeleton attached to focal adhesions. We measured contractile forces using two-dimensional traction microscopy (Fig. 4A) and characterized the contractility of each cell by assessing the elastic strain energy stored in the ECM (Fig. 4B). In general, keratinocytes displayed significantly higher strain energy compared to myoblasts (Fig. 4C). These findings support the notion that higher cellular stiffness (as observed in keratinocytes compared to myoblasts) correlates with higher contractile forces. Moreover, the strain energy of plectin^−/−^ myoblasts was around threefold lower (0.6 pJ) than that of plectin^+/+^ myoblasts (1.7 pJ), whereas a higher strain energy of plectin^−/−^ (3.6 pJ) compared to plectin^+/+^ keratinocytes (2.9 pJ) was observed.

### Plectin-deficiency influences cell motility in keratinocytes, but not in myoblasts

**Fig. 5:**
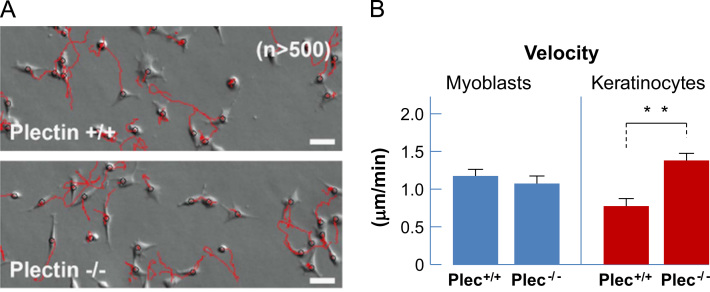
Analyses of cell motility. (A) Representative images from time lapse movies over 5 h. In red, the trajectories of individual myoblasts are displayed. Bars, 20 µm. (B) Statistical analysis of cell motility. The mean square displacement (MSD) was determined from trajectories of plectin^−/−^ and plectin^+/+^ cells. For each cell line, the trajectories of more than 500 individual cells were evaluated; the data represent mean±SE. ^⁎⁎^*p*<0.01 from unpaired Student׳s *t-*test.

To investigate the influence of plectin-deficiency on two-dimensional cell motility, we analyzed all cell lines on collagen type I-coated glass coverslips (Fig. 5A). While the migration speed for plectin^−/−^ and plectin^+/+^ myoblasts was comparable, the velocity in plectin^−/−^ keratinocytes increased significantly compared to plectin^+/+^ keratinocytes (Fig. 5B), which is consistent with previous results [17].

## Discussion

Plectin׳s function has been extensively studied using a combination of in vitro assays, mouse models, and cell lines, with special emphasis on plectin isoforms, plectin binding partners and IF network formation (for reviews, see [1–4,6]). Major findings were directed to the function of plectin as a general plasma membrane-associated cytoskeleton organizer involved in the shape formation and polarization of cells. Moreover, plectin plays an important role as a cytolinker and scaffolding platform for the assembly of protein complexes which are involved in cellular signaling and cytoskeleton dynamics. For instance, plectin deficiency in cells has been reported to affect *rho, rac* and *cdc42* GTPase activities leading to reduced actin filament dynamics and influencing MAP kinase cascades with consequences for cell migration and stress responses [17–21]. Plectin also influences the stability and dynamics of microtubules by antagonizing the stabilizing function of MAP kinase [22]. In essence, plectin is a central player for many cellular processes that require cytoskeletal restructuring and reorganization involving all three major cytoskeletal filament network systems (IFs, actin filaments, and microtubules). Based on data from a variety of cell systems as well as from EBS-MD patients, it is becoming increasingly clear that plectin dysfunction affects cells most when they have to respond to stress.

Most mutations in the human plectin gene (PLEC) cause autosomal recessive EBS-MD showing features of severe skin blistering and progressive muscular dystrophy. Moreover, PLEC mutations can cause EBS-MD associated with a myasthenic syndrome (EBS-MD-MyS), EBS with pyloric atresia (EBS-PA), EBS-Ogna, or limb-girdle muscular dystrophy type 2Q (LGMD2Q). All plectin-related diseases are characterized by either muscular dystrophy or skin fragility, or a combination of both [4]. As skin and skeletal muscle are obviously the most affected tissues in plectinopathic patients, we compared in this study the biomechanical properties and intrinsic mechanical stress responses of murine plectin^−/−^ keratinocytes and myoblasts to the respective wild-type cells.

In our study, we combined a unique collection of biomechanical methods (cell stretching, magnetic tweezer microrheology, traction force microscopy, and motility assays) to determine the mechanical effects of plectin in cells. We observed that wild-type myoblasts and keratinocytes show marked differences in their response to the application of external stress. Keratinocytes were generally more vulnerable in response to external mechanical stress, stiffer, and more contractile, less adhesive and less fluid, as well as less motile compared to myoblasts.

Plectin-deficiency caused diverging biomechanical responses in myoblasts vs. keratinocytes (Table 1). The lack of plectin decreased the stress vulnerability, cell stiffness, adhesion strength, and strain energy in myoblasts, while it increased cell stiffness, adhesion strength, strain energy, and motility in keratinocytes [17,23,24]. Cytoskeletal dynamics (fluidity) was increased in plectin^−/−^ myoblasts, while it remained unchanged in keratinocytes.

In both, myoblasts and keratinocytes, structural IF network alterations upon plectin deficiency have been reported, which are consistent with the diverging stiffness changes we found in our study (Fig. 2C). In plectin^−/−^ keratinocytes, keratin filaments were more bundled and less flexible compared to wild-type cells, which led to IF networks of greater mesh size [1,17]. In addition, microtubules were reported to be more stable [17,22,23]. No such obvious alterations were observed for desmin networks in undifferentiated plectin^−/−^ myoblasts; however, large desmin-positive protein aggregates appeared upon differentiation into multinucleated myotubes [7]. The total amount of IF proteins, i.e. keratin in plectin^−/−^ keratinocytes and desmin in plectin^−/−^ myoblasts, is not changed to wild-type cells according to Refs. [17] and [7].

Our observations provide clear evidence that plectin fulfills distinct biomechanical functions in different cell types. Recent studies have focused on the pathological consequences of cells lacking plectin, thus exerting a dominant negative effect on the ordered formation of the IF network system, consecutively leading to IF network collapse and abnormal protein aggregation. This is the case during myoblast differentiation and skeletal muscle formation, but only occurs upon external stress (hyperphosphorylation, nitrosylation) in keratinocytes [7,17,25]. From our limited data, it is tempting to speculate that the decreased biomechanics (lower contractility, adhesion strength and cytoskeletal stability) of plectin^−/−^ myoblasts might directly or indirectly contribute to the progressive muscle degeneration and weakness. However, these factors are at the same time protective when cells are externally stressed, as observed by the decreased stress vulnerability of pectin^−/−^ myoblasts. Thus, the biomechanical analysis of individual cells alone can neither provide ultimate answers to the underlying disease cause of EBS-MD nor comprehensively explain its progressive nature. Nonetheless, our study demonstrates that plectin has complex and divergent biomechanical implications in different cell types and tissues.

## Figures and Tables

**Table 1:** Summary of changes in biomechanical properties of plectin^−/−^ myoblasts and keratinocytes compared to plectin^+/+^ cells.

**Biomechanical analysis**	**Myoblasts**	**Keratinocytes**
**Vulnerability**	↓	↔
**Cell stiffness**	↓	↑
**Adhesion (Binding) strength**	↓	↑
**Fluidity (Dynamics)**	↑	↔
**Strain energy**	↓	↑
**Velocity**	↔	↑

↑, increase compared to plectin^+/+^ cells; ↓, decrease compared to plectin^+/+^ cells; ↔, comparable to plectin^+/+^ cells.

## References

[1] Wiche G., Winter L. (2011). Plectin isoforms as organizers of intermediate filament cytoarchitecture. Bioarchitecture.

[2] Castañón M.J., Walko G., Winter L., Wiche G. (2013). Plectin-intermediate filament partnership in skin, skeletal muscle, and peripheral nerve. Histochem. Cell Biol..

[3] Rezniczek G.A., Walko G., Wiche G. (2010). Plectin gene defects lead to various forms of epidermolysis bullosa simplex. Dermatol. Clin..

[4] Winter L., Wiche G. (2013). The many faces of plectin and plectinopathies: pathology and mechanisms. Acta. Neuropathol..

[5] Konieczny P., Fuchs P., Reipert S., Kunz W.S., Zeold A., Fischer I., Paulin D., Schröder R., Wiche G. (2008). Myofiber integrity depends on desmin network targeting to Z-disks and costameres via distinct plectin isoforms. J. Cell Biol..

[6] Konieczny P., Wiche G. (2008). Muscular integrity--a matter of interlinking distinct structures via plectin. Adv. Exp. Med. Biol..

[7] Winter L., Staszewska I., Mihailovska E., Fischer I., Goldmann W.H., Schröder R., Wiche G. (2014). Chemical chaperone ameliorates pathological protein aggregation in plectin-deficient muscle. J. Clin. Invest..

[8] Andrä K., Kornacker I., Jorgl A., Zorer M., Spazierer D., Fuchs P., Fischer I., Wiche G. (2003). Plectin-isoform-specific rescue of hemidesmosomal defects in plectin (−/−) keratinocytes. J. Invest. Dermatol..

[9] Faust U., Hampe N., Rubner W., Kirchgessner N., Safran S., Hoffmann B., Merkel R. (2011). Cyclic stress at mHz frequencies aligns fibroblasts in direction of zero strain. PLoS One.

[10] Bonakdar N., Lautscham L.A., Czonstke M., Koch T.M., Mainka A., Jungbauer T., Goldmann W.H., Schröder R., Fabry B. (2012). Biomechanical characterization of a desminopathy in primary human myoblasts. Biochem. Biophys. Res. Commun..

[11] Kollmannsberger P., Fabry B. (2007). High-force magnetic tweezers with force feedback for biological applications. Rev. Sci. Instrum..

[12] Pelham R.J., Wang Y.L. (1998). Cell locomotion and focal adhesions are regulated by the mechanical properties of the substrate. Biol. Bull. 194.

[13] Raupach C., Zitterbart D.P., Mierke C.T., Metzner C., Müller F.A., Fabry B. (2007). Stress fluctuations and motion of cytoskeletal-bound markers. Phys. Rev. E. Stat. Nonlin. Soft Matter. Phys..

[14] Butler J.P., Tolic-Norrelykke I.M., Fabry B., Fredberg J.J. (2002). Traction fields, moments, and strain energy that cells exert on their surroundings. Am. J. Physiol. Cell Physiol..

[15] Kollmannsberger P., Mierke C.T., Fabry B. (2011). Nonlinear viscoelasticity of adherent cells is controlled by cytoskeletal tension. Soft Matter..

[16] Bursac P., Lenormand G., Fabry B., Oliver M., Weitz D.A., Viasnoff V., Butler J.P., Fredberg J.J. (2005). Cytoskeletal remodelling and slow dynamics in the living cell. Nat. Mater..

[17] Osmanagic-Myers S., Gregor M., Walko G., Burgstaller G., Reipert S., Wiche G. (2006). Plectin-controlled keratin cytoarchitecture affects MAP kinases involved in cellular stress response and migration. J. Cell Biol..

[18] Burgstaller G., Gregor M., Winter L., Wiche G. (2010). Keeping the vimentin network under control: cell-matrix adhesion-associated plectin 1f affects cell shape and polarity of fibroblasts. Mol. Biol. Cell.

[19] Gregor M., Zeöld A., Oehler S., Marobela K.A., Fuchs P., Weigel G., Hardie D.G., Wiche G. (2006). Plectin scaffolds recruit energy-controlling AMP-activated protein kinase (AMPK) in differentiated myofibres. J. Cell Sci..

[20] Na S., Chowdhury F., Tay B., Ouyang M., Gregor M., Wang Y., Wiche G., Wang N. (2009). Plectin contributes to mechanical properties of living cells. Am. J. Physiol. Cell Physiol..

[21] Andrä K., Nikolic B., Stocher M., Drenckhahn D., Wiche G. (1998). Not just scaffolding: plectin regulates actin dynamics in cultured cells. Genes Dev..

[22] Valencia R.G., Walko G., Janda L., Novacek J., Mihailovska E., Reipert S., Andrä-Marobela K., Wiche G. (2013). Intermediate filament-associated cytolinker plectin 1c destabilizes microtubules in keratinocytes. Mol. Biol. Cell.

[23] Seltmann K., Fritsch A.W., Käs J.A., Magin T.M. (2013). Keratins significantly contribute to cell stiffness and impact invasive behavior. Proc. Natl. Acad. Sci. USA.

[24] Seltmann K., Roth W, Kröger C, Loschke F, Lederer M, Hüttelmaier S, Magin T.M. (2013). Keratins mediate localization of hemidesmosomes and repress cell motility. J. Invest. Dermatol..

[25] Spurny R., Abdoulrahman K., Janda L., Rünzler D., Köhler G., Castañón M.J., Wiche G. (2007). Oxidation and nitrosylation of cysteines proximal to the intermediate filament (IF)-binding site of plectin: effects on structure and vimentin binding and involvement in IF collapse. J. Biol. Chem..

